# Bis(4-amino-2-chloro­phen­yl) disulfide

**DOI:** 10.1107/S1600536811014425

**Published:** 2011-04-22

**Authors:** Jun-Mei Tang, Zhi-Qiang Feng, Wei Cheng

**Affiliations:** a Department of Biological and Chemical Engineering, Chien-shiung Institute of Technology, Taicang 215411, Suzhou, People’s Republic of China; b School of Materials Engineering, Jinling Institute of Technology, Nanjing 211169, People’s Republic of China

## Abstract

The title compound, C_12_H_10_Cl_2_N_2_S_2_, features an S—S bond [2.0671 (16) Å] that bridges two 4-amino-2-chloro­phenyl rings with a C—S—S—C torsion angle of −84.2 (2)°. The two benzene rings are twisted with respect to each other at a dihedral angle of 39.9 (2)°. Inter­molecular N—H⋯S hydrogen bonding is present in the crystal structure.

## Related literature

For the application of the title compound, see: Crowley (1964 ▶). For S—S bond distances, see: Allen *et al.* (1991 ▶). For similar C—S—S—C torsion angles in disulfide compounds, see: Korp & Bernal (1984 ▶); Poveteva & Zvonkova (1975 ▶).
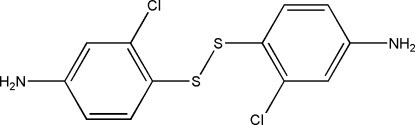


## Experimental

### 

#### Crystal data

C_12_H_10_Cl_2_N_2_S_2_
*M* *_r_* = 317.24
Monoclinic, 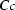

*a* = 6.6360 (13) Å
*b* = 14.907 (3) Å
*c* = 13.588 (3) Å
β = 95.09 (3)°
*V* = 1338.9 (5) Å^3^
*Z* = 4
Mo *K*α radiation
μ = 0.78 mm^−1^
*T* = 296 K
0.30 × 0.20 × 0.10 mm

#### Data collection

Enraf–Nonius CAD-4 diffractometer
Absorption correction: ψ scan (North *et al.*, 1968 ▶) *T*_min_ = 0.800, *T*_max_ = 0.940
2606 measured reflections
1331 independent reflections
1221 reflections with *I* > 2σ(*I*)
*R*_int_ = 0.026
3 standard reflections every 200 reflections  intensity decay: 1%

#### Refinement

*R*[*F* ^2^ > 2σ(*F* ^2^)] = 0.031
*wR*(*F* ^2^) = 0.088
*S* = 1.00
1331 reflections
163 parameters
2 restraints
H-atom parameters constrained
Δρ_max_ = 0.19 e Å^−3^
Δρ_min_ = −0.22 e Å^−3^
Absolute structure: Flack (1983 ▶), 110 Friedel parirs
Flack parameter: 0.09 (11)

### 

Data collection: *CAD-4 Software* (Enraf–Nonius, 1985 ▶); cell refinement: *CAD-4 Software*; data reduction: *XCAD4* (Harms & Wocadlo, 1995 ▶); program(s) used to solve structure: *SHELXTL* (Sheldrick, 2008 ▶); program(s) used to refine structure: *SHELXTL*; molecular graphics: *SHELXTL*; software used to prepare material for publication: *SHELXTL*.

## Supplementary Material

Crystal structure: contains datablocks I, global. DOI: 10.1107/S1600536811014425/xu5174sup1.cif

Structure factors: contains datablocks I. DOI: 10.1107/S1600536811014425/xu5174Isup2.hkl

Additional supplementary materials:  crystallographic information; 3D view; checkCIF report

## Figures and Tables

**Table 1:** Hydrogen-bond geometry (Å, °)

*D*—H⋯*A*	*D*—H	H⋯*A*	*D*⋯*A*	*D*—H⋯*A*
N1—H1*A*⋯S1^i^	0.86	2.80	3.611 (5)	158
N2—H2*A*⋯S2^ii^	0.86	2.86	3.684 (5)	162

Symmetry codes: (i) 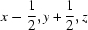; (ii) 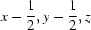.

**Fig. 1.**
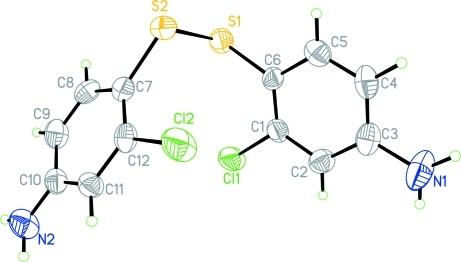
The structure of the molecule of (I). Displacement ellipsoids are drawn at the 50% probability level.

## supplementary crystallographic information

### Comment

The title compound has been used as fungicide and mildew-proofing agent (Crowley, 1964). We herein report its crystal structure. The S-S distance, 2.0670 (13)Å, is normal and falls within the range of 2.018-2.099Å found for the acyclic disulfides in the Cambridge Structural Database (Allen *et al.*, 1991). The torsion angle C-S-S-C of 84.2 (2)° is close to the 85.0° found in diphenyldisulfide (Korp & Bernal, 1984) and lower than the 101.7° found in 4-amino-4'-nitrodiphenyl disulfide (Poveteva & Zvonkova, 1975). The intermolecular N–H···S hydrogen bonds may be effective in the stabilization of the crystal structure.

### Experimental

The aqueous solution (20 ml) of 3,4-dichloronitrobenzene (19.2 g, 0.1 mol) and sodium sulfhydrate (28.5 g, 0.22 mol) was refluxed for 16 h, and then filtered. The title compound was obtained from the filtrate. The single crystals were obtained by recrystallization from an ethanol solution after 5 d.

### Refinement

H atoms were positioned geometrically with N—H = 0.86 and C—H = 0.93 Å, and constrained to ride on their parent atoms with *U*_iso_(H) = 1.2*U*_eq_(C,N). As a half of reciprocal space diffraction data were collected only using a four-circle diffractometer, Friedel pair coverage is low in this determination.

### Crystal data

**Table:**

C_12_H_10_Cl_2_N_2_S_2_	*F*(000) = 648
*M**_r_* = 317.24	*D*_x_ = 1.574 Mg m^−^^3^
Monoclinic, *C**c*	Mo *K*α radiation, λ = 0.71073 Å
Hall symbol: C -2yc	Cell parameters from 25 reflections
*a* = 6.6360 (13) Å	θ = 10–14°
*b* = 14.907 (3) Å	µ = 0.78 mm^−^^1^
*c* = 13.588 (3) Å	*T* = 296 K
β = 95.09 (3)°	Block, yellow
*V* = 1338.9 (5) Å^3^	0.30 × 0.20 × 0.10 mm
*Z* = 4	

### Data collection

**Table:**

Enraf–Nonius CAD-4 diffractometer	1221 reflections with *I* > 2σ(*I*)
Radiation source: fine-focus sealed tube	*R*_int_ = 0.026
graphite	θ_max_ = 25.4°, θ_min_ = 2.7°
ω/2θ scans	*h* = 0→7
Absorption correction: ψ scan (North *et al.*, 1968)	*k* = −17→17
*T*_min_ = 0.800, *T*_max_ = 0.940	*l* = −16→16
2606 measured reflections	3 standard reflections every 200 reflections
1331 independent reflections	intensity decay: 1%

### Refinement

**Table:**

Refinement on *F*^2^	Secondary atom site location: difference Fourier map
Least-squares matrix: full	Hydrogen site location: inferred from neighbouring sites
*R*[*F*^2^ > 2σ(*F*^2^)] = 0.031	H-atom parameters constrained
*wR*(*F*^2^) = 0.088	*w* = 1/[σ^2^(*F*_o_^2^) + (0.066*P*)^2^] where *P* = (*F*_o_^2^ + 2*F*_c_^2^)/3
*S* = 1.00	(Δ/σ)_max_ < 0.001
1331 reflections	Δρ_max_ = 0.19 e Å^−^^3^
163 parameters	Δρ_min_ = −0.22 e Å^−^^3^
2 restraints	Absolute structure: Flack (1983), 110 Friedel parirs
Primary atom site location: structure-invariant direct methods	Flack parameter: 0.09 (11)

### Special details

**Table:**

Geometry. All esds (except the esd in the dihedral angle between two l.s. planes) are estimated using the full covariance matrix. The cell esds are taken into account individually in the estimation of esds in distances, angles and torsion angles; correlations between esds in cell parameters are only used when they are defined by crystal symmetry. An approximate (isotropic) treatment of cell esds is used for estimating esds involving l.s. planes.
Refinement. Refinement of F^2^ against ALL reflections. The weighted R-factor wR and goodness of fit S are based on F^2^, conventional R-factors R are based on F, with F set to zero for negative F^2^. The threshold expression of F^2^ > 2sigma(F^2^) is used only for calculating R-factors(gt) etc. and is not relevant to the choice of reflections for refinement. R-factors based on F^2^ are statistically about twice as large as those based on F, and R- factors based on ALL data will be even larger.

### Fractional atomic coordinates and isotropic or equivalent isotropic displacement parameters (Å^2^)

**Table:**

	*x*	*y*	*z*	*U*_iso_*/*U*_eq_	
S1	0.28662 (15)	0.23231 (7)	0.80302 (8)	0.0442 (3)	
Cl1	−0.1503 (2)	0.18083 (8)	0.87818 (12)	0.0609 (4)	
N1	−0.3017 (9)	0.5116 (3)	0.9000 (3)	0.0681 (14)	
H1A	−0.2710	0.5674	0.8950	0.082*	
H1B	−0.4135	0.4965	0.9229	0.082*	
C1	−0.0811 (7)	0.2917 (3)	0.8603 (3)	0.0380 (9)	
Cl2	−0.2000 (2)	0.26890 (7)	0.57856 (11)	0.0586 (4)	
S2	0.26156 (17)	0.21357 (8)	0.65170 (9)	0.0441 (3)	
N2	−0.3804 (7)	−0.0596 (3)	0.5629 (3)	0.0525 (10)	
H2A	−0.3514	−0.1157	0.5684	0.063*	
H2B	−0.5012	−0.0431	0.5426	0.063*	
C2	−0.2194 (8)	0.3561 (3)	0.8835 (3)	0.0431 (10)	
H2C	−0.3415	0.3397	0.9070	0.052*	
C3	−0.1716 (8)	0.4459 (3)	0.8708 (3)	0.0453 (11)	
C4	0.0098 (8)	0.4693 (3)	0.8332 (3)	0.0455 (11)	
H4A	0.0401	0.5292	0.8226	0.055*	
C5	0.1417 (8)	0.4043 (3)	0.8121 (3)	0.0425 (10)	
H5A	0.2634	0.4208	0.7881	0.051*	
C6	0.1012 (6)	0.3126 (3)	0.8252 (3)	0.0358 (9)	
C7	0.0675 (7)	0.1343 (3)	0.6287 (3)	0.0364 (9)	
C8	0.1075 (7)	0.0430 (3)	0.6402 (3)	0.0418 (10)	
H8A	0.2376	0.0250	0.6627	0.050*	
C9	−0.0379 (8)	−0.0212 (3)	0.6193 (3)	0.0464 (11)	
H9A	−0.0047	−0.0815	0.6277	0.056*	
C10	−0.2349 (7)	0.0029 (3)	0.5857 (3)	0.0379 (10)	
C11	−0.2776 (7)	0.0940 (3)	0.5730 (3)	0.0371 (9)	
H11A	−0.4068	0.1122	0.5490	0.044*	
C12	−0.1295 (7)	0.1569 (3)	0.5957 (3)	0.0377 (10)	

### Atomic displacement parameters (Å^2^)

**Table:**

	*U*^11^	*U*^22^	*U*^33^	*U*^12^	*U*^13^	*U*^23^
S1	0.0341 (6)	0.0425 (6)	0.0543 (6)	0.0054 (5)	−0.0052 (5)	−0.0064 (5)
Cl1	0.0460 (6)	0.0332 (5)	0.1040 (10)	−0.0052 (5)	0.0090 (6)	0.0041 (6)
N1	0.088 (4)	0.049 (2)	0.071 (3)	0.026 (3)	0.027 (3)	0.007 (2)
C1	0.036 (2)	0.031 (2)	0.046 (2)	0.0004 (18)	−0.0038 (19)	−0.0030 (17)
Cl2	0.0485 (7)	0.0276 (5)	0.0974 (10)	0.0017 (5)	−0.0065 (6)	0.0012 (6)
S2	0.0369 (6)	0.0427 (6)	0.0538 (6)	−0.0042 (5)	0.0115 (5)	−0.0047 (5)
N2	0.057 (2)	0.0300 (19)	0.069 (3)	−0.0092 (18)	−0.001 (2)	0.0023 (18)
C2	0.038 (2)	0.044 (2)	0.048 (2)	0.003 (2)	0.0039 (19)	0.003 (2)
C3	0.060 (3)	0.039 (2)	0.036 (2)	0.011 (2)	0.000 (2)	0.0018 (19)
C4	0.065 (3)	0.030 (2)	0.042 (2)	0.002 (2)	0.003 (2)	0.0003 (18)
C5	0.046 (3)	0.041 (2)	0.040 (2)	−0.007 (2)	0.003 (2)	0.0019 (18)
C6	0.030 (2)	0.033 (2)	0.043 (2)	0.0057 (17)	−0.0024 (17)	−0.0062 (16)
C7	0.037 (2)	0.035 (2)	0.038 (2)	0.0007 (18)	0.0072 (17)	−0.0044 (17)
C8	0.036 (2)	0.044 (2)	0.047 (2)	0.012 (2)	0.0091 (19)	0.0015 (19)
C9	0.060 (3)	0.028 (2)	0.051 (3)	0.005 (2)	0.006 (2)	−0.0003 (17)
C10	0.043 (3)	0.036 (2)	0.034 (2)	−0.0029 (18)	0.005 (2)	−0.0024 (17)
C11	0.035 (2)	0.034 (2)	0.042 (2)	−0.0049 (17)	0.0024 (18)	−0.0014 (17)
C12	0.048 (3)	0.0219 (19)	0.043 (2)	0.0055 (19)	0.0043 (19)	−0.0025 (16)

### Geometric parameters (Å, °)

**Table:**

S1—C6	1.762 (4)	C3—C4	1.393 (7)
S1—S2	2.0671 (16)	C4—C5	1.354 (7)
Cl1—C1	1.738 (4)	C4—H4A	0.9300
N1—C3	1.387 (6)	C5—C6	1.407 (6)
N1—H1A	0.8601	C5—H5A	0.9300
N1—H1B	0.8599	C7—C12	1.385 (7)
C1—C6	1.375 (7)	C7—C8	1.393 (6)
C1—C2	1.384 (6)	C8—C9	1.371 (7)
Cl2—C12	1.744 (4)	C8—H8A	0.9300
S2—C7	1.755 (4)	C9—C10	1.393 (7)
N2—C10	1.357 (6)	C9—H9A	0.9300
N2—H2A	0.8599	C10—C11	1.396 (6)
N2—H2B	0.8600	C11—C12	1.374 (7)
C2—C3	1.390 (6)	C11—H11A	0.9300
C2—H2C	0.9300		
			
C6—S1—S2	105.43 (14)	C6—C5—H5A	118.9
C3—N1—H1A	120.2	C1—C6—C5	116.6 (4)
C3—N1—H1B	119.8	C1—C6—S1	123.7 (3)
H1A—N1—H1B	120.0	C5—C6—S1	119.6 (4)
C6—C1—C2	122.9 (4)	C12—C7—C8	116.0 (4)
C6—C1—Cl1	121.0 (3)	C12—C7—S2	123.4 (3)
C2—C1—Cl1	116.1 (4)	C8—C7—S2	120.5 (4)
C7—S2—S1	105.13 (15)	C9—C8—C7	122.4 (4)
C10—N2—H2A	119.9	C9—C8—H8A	118.8
C10—N2—H2B	120.1	C7—C8—H8A	118.8
H2A—N2—H2B	120.0	C8—C9—C10	120.7 (4)
C1—C2—C3	118.5 (5)	C8—C9—H9A	119.7
C1—C2—H2C	120.8	C10—C9—H9A	119.7
C3—C2—H2C	120.8	N2—C10—C9	121.7 (4)
N1—C3—C2	119.4 (5)	N2—C10—C11	120.5 (4)
N1—C3—C4	120.6 (4)	C9—C10—C11	117.8 (4)
C2—C3—C4	120.0 (4)	C12—C11—C10	120.2 (4)
C5—C4—C3	119.7 (4)	C12—C11—H11A	119.9
C5—C4—H4A	120.2	C10—C11—H11A	119.9
C3—C4—H4A	120.2	C11—C12—C7	122.9 (4)
C4—C5—C6	122.3 (5)	C11—C12—Cl2	116.4 (4)
C4—C5—H5A	118.9	C7—C12—Cl2	120.7 (3)
			
C6—S1—S2—C7	−84.2 (2)	S1—S2—C7—C12	99.7 (4)
C6—C1—C2—C3	−0.1 (7)	S1—S2—C7—C8	−82.5 (3)
Cl1—C1—C2—C3	179.9 (4)	C12—C7—C8—C9	0.6 (6)
C1—C2—C3—N1	−175.6 (4)	S2—C7—C8—C9	−177.4 (3)
C1—C2—C3—C4	1.6 (7)	C7—C8—C9—C10	−0.2 (6)
N1—C3—C4—C5	175.0 (4)	C8—C9—C10—N2	178.8 (4)
C2—C3—C4—C5	−2.1 (7)	C8—C9—C10—C11	0.8 (6)
C3—C4—C5—C6	1.1 (6)	N2—C10—C11—C12	−179.7 (4)
C2—C1—C6—C5	−0.8 (6)	C9—C10—C11—C12	−1.7 (6)
Cl1—C1—C6—C5	179.2 (3)	C10—C11—C12—C7	2.1 (6)
C2—C1—C6—S1	175.6 (3)	C10—C11—C12—Cl2	−179.2 (3)
Cl1—C1—C6—S1	−4.4 (5)	C8—C7—C12—C11	−1.5 (6)
C4—C5—C6—C1	0.3 (6)	S2—C7—C12—C11	176.4 (3)
C4—C5—C6—S1	−176.3 (3)	C8—C7—C12—Cl2	179.8 (3)
S2—S1—C6—C1	102.4 (3)	S2—C7—C12—Cl2	−2.2 (5)
S2—S1—C6—C5	−81.3 (3)		

### Hydrogen-bond geometry (Å, °)

**Table:**

*D*—H···*A*	*D*—H	H···*A*	*D*···*A*	*D*—H···*A*
N1—H1A···S1^i^	0.86	2.80	3.611 (5)	158
N2—H2A···S2^ii^	0.86	2.86	3.684 (5)	162

Symmetry codes: (i) *x*−1/2, *y*+1/2, *z*; (ii) *x*−1/2, *y*−1/2, *z*.

## References

[bb1] Allen, F. H., Davies, J. E., Galloy, J. J., Johnson, O., Kennard, O., Macrae, C. F., Mitchell, E. M., Mitchell, G. F., Smith, J. M. & Watson, D. G. (1991). *J. Chem. Inf. Comput. Sci.* **31**, 187–204.

[bb2] Crowley, D. J. (1964). US Patent No. 3 150 186.

[bb3] Enraf–Nonius (1985). *CAD-4 Software* Enraf–Nonius, Delft, The Netherlands.

[bb4] Flack, H. D. (1983). *Acta Cryst.* A**39**, 876–881.

[bb5] Harms, K. & Wocadlo, S. (1995). *XCAD4* University of Marburg, Germany.

[bb6] Korp, J. D. & Bernal, I. (1984). *J. Mol. Struct.* **118**, 157–164.

[bb7] North, A. C. T., Phillips, D. C. & Mathews, F. S. (1968). *Acta Cryst.* A**24**, 351–359.

[bb8] Poveteva, Z. P. & Zvonkova, Z. V. (1975). *Kristallografiya*, **20**, 69–73.

[bb9] Sheldrick, G. M. (2008). *Acta Cryst.* A**64**, 112–122. 10.1107/S0108767307043930 18156677

